# Physical activity from young adulthood to middle age and premature cardiovascular disease events: a 30-year population-based cohort study

**DOI:** 10.1186/s12966-022-01357-2

**Published:** 2022-09-20

**Authors:** Jason M. Nagata, Eric Vittinghoff, Kelley Pettee Gabriel, Jamal S. Rana, Andrea K. Garber, Andrew E. Moran, Jared P. Reis, Cora E. Lewis, Stephen Sidney, Kirsten Bibbins-Domingo

**Affiliations:** 1 Division of Adolescent and Young Adult Medicine, Department of Pediatrics, University of California, 550 16th Street, 4th Floor, Box 0110, San Francisco, California 94158 USA; 2 Department of Epidemiology and Biostatistics, University of California, San Francisco, CA USA; 3 Department of Epidemiology, University of Alabama at Birmingham, Birmingham, AL USA; 4 Division of Cardiology, Kaiser Permanente Northern California, Oakland, CA USA; 5 Division of Research, Kaiser Permanente Northern California, Oakland, CA USA; 6 Division of General Medicine, Columbia University, New York, NY USA; 7 Division of Cardiovascular Sciences, National Heart, Lung, and Blood Institute, Bethesda, MD USA

**Keywords:** Physical activity, Exercise, Cardiovascular disease, Stroke, Heart failure

## Abstract

**Background:**

Although physical activity is generally protective of cardiovascular disease (CVD), less is known about how young adult physical activity relates to premature CVD events. The objective of this study was to determine the association between level and change in physical activity from young adulthood to middle age and incidence of premature CVD events before age 60.

**Methods:**

We analyzed data collected across four urban sites from nine visits over 30 years of follow-up (1985–2016) from the Coronary Artery Risk Development in Young Adults (CARDIA) study, a prospective community-based cohort study of 5115 Black and White women and men aged 18–30 years at baseline (1985–1986). Linear mixed models were used to develop individualized moderate-to-vigorous intensity self-reported physical activity trajectories per participant. Fatal and nonfatal coronary heart disease (CHD), heart failure, and stroke outcomes were analyzed separately and as a combined CVD event outcome.

**Results:**

Overall, physical activity declined in young adults as they progressed through middle age. Lower physical activity scores (per 100 exercise units) in 18 year-olds were associated with higher odds of premature CHD (AOR 1.14, 95% CI 1.02–1.28), heart failure (AOR 1.21, 95% CI 1.05–1.38), stroke (AOR 1.20, 95% CI 1.04–1.39), and any CVD (AOR 1.15, 95% CI 1.06–1.24) events. Each additional annual 1-unit reduction in the physical activity score was associated with a higher annual odds of incident heart failure (1.07, 95% CI 1.02–1.13), stroke (1.06, 95% CI 1.00–1.13), and CVD (1.04, 95% CI 1.01–1.07) events. Meeting the minimum (AOR 0.74, 95% CI 0.0.57–0.96) and twice the minimum (AOR 0.55, 95% CI 0.34–0.91) Department of Health and Human Services physical activity guidelines through follow up was protective of premature CVD events.

**Conclusions:**

Given recent trends in declining physical activity with age and associated premature CVD events, the transition from young adult to midlife is an important time period to promote physical activity.

**Supplementary Information:**

The online version contains supplementary material available at 10.1186/s12966-022-01357-2.

## Introduction

Cardiovascular disease (CVD) is the leading cause of death in the United States (US), with over 130 million adults in the US (45.1%) projected to have CVD by 2035 [1]. Despite prior studies noting the relationship between physical activity and CVD events (hospitalizations and mortality) [2–4], the 2018 Department of Health and Human Services (HHS) Physical Activity Guidelines noted important research gaps of public health importance [5]. The HHS Guidelines determined that there was insufficient evidence to determine if the relationship between physical activity and CVD events vary by age. Thus, further research investigating the importance of timing of the physical activity exposure across the life course is needed [5]. For instance, little is known about how physical activity trajectories (level and annual change) from young adulthood to middle age (approximately 18 to 65 years) influence CVD events, particularly premature events prior to age 60.

Serious gaps remain in our understanding of recommended levels of physical activity during young adulthood [5]. Young adulthood is an important transition period that sets physical activity patterns for the rest of adulthood and may represent a critical window for intervention [6–8]. On average, physical activity declines markedly during young adulthood, when individuals often transition from high school to college or the workforce [6–8]. The Physical Activity Guidelines noted young adults have unique growth and developmental needs similar to adolescents [5]. However, the optimal dose of physical activity, particularly in young adulthood, to prevent CVD events, remains unknown [9, 10]. Furthermore, physical activity disparities by race and sex have been noted in young adulthood and through the adult life course [11, 12], but it is unknown if race or sex modifies the effect of physical activity and CVD events.

We sought to quantify the impact of young adult and midlife physical activity on metabolic risk factors by analyzing data from the Coronary Artery Risk Development in Young Adults (CARDIA) Study, a large prospective cohort starting in young adulthood with 30 years of follow-up. CARDIA collected repeated measures of moderate and vigorous intensity physical activity, which allow for the development of individual trajectories of physical activity from young adulthood through middle age. The objective of this study was to determine the independent associations between young adult level of physical activity and subsequent changes in physical activity through the transition to midlife and incidence of premature CVD events (coronary heart disease [CHD], heart failure, stroke). In addition, we examined if meeting the current adult aerobic physical activity guideline levels were protective of premature CVD events. We hypothesized low physical activity levels at age 18 and declines in physical activity through the adult life course will be associated with incidence of premature CVD events.

## Methods

### Study population

The CARDIA Study recruited Black and White young adults (*N* = 5115) from four urban sites (Birmingham, Alabama; Chicago, Illinois; Minneapolis, Minnesota; and Oakland, California). Baseline data collection occurred in 1985–1986 and participants have been followed up for more than 30 years with high retention. Retention was 91, 86, 81, 77, 74, 72, 72, and 71% at years 2, 5, 7, 10, 15, 20, 25, 30, respectively. At baseline, the cohort was designed to be approximately balanced within center by age (18–24 years and 25–30 years), race (Black and White), sex (male and female), and educational level (high school or less or higher than high school). Following the baseline examination, one participant requested to be excluded from further analyses. Additional details about the study design are described elsewhere [13]. The institutional review boards at each study site approved study procedures. All participants provided written informed consent.

### Measures

#### Physical activity

Physical activity was ascertained by the interviewer-administered CARDIA Physical Activity History Questionnaire at each of the 9 examinations [14, 15]. Participants self-reported their frequency of participation in 13 activity categories (8 vigorous and 5 moderate intensity) within time for leisure and occupational physical activity domains over the past 12 months. Each activity’s intensity was measured as metabolic equivalents of task (METs), where 1 MET is the energy expended at rest (an approximate oxygen consumption of 3.5 mL/1 kg of body weight/minute). Vigorous intensity activities (≥6 METs) included running, swimming, heavy lifting, and vigorous exercise classes. Moderate intensity activities (3–5.9 METs) included walking, non-strenuous sports (e.g. softball), and home maintenance (e.g., raking, gardening) [16]. A frequency was assigned to each activity based on the following criteria: 1) whether it was performed for ≥1 hour or during any 1 month in the past year, 2) the number of months it was performed at that level, and 3) the number of months the activity was performed frequently. Duration thresholds (2–5 hours per week) and intensity scores (3–8 METs) were assigned to each activity. Frequent participation was defined as above these levels [15]. Frequency (number of months) of participation was multiplied by the intensity (METs) of each activity, with a weighting factor for the months of more frequent participation, to calculate the moderate-to-vigorous-intensity physical activity score [17]. All activities were summed to estimate the total activity score expressed in exercise units (EUs). For reference, a physical activity score of 300 EU approximates the HHS recommendations of approximately 150 minutes of moderate-intensity activity per week [18, 19]. Convergent validity of the CARDIA Physical Activity History questionnaire has been established using report-based measures, including physical activity diaries and detailed quantitative recall questionnaires [14, 17, 20] and accelerometers [20–22]. The CARDIA physical activity questionnaire demonstrated test-retest reliability of 0.77–0.84 [17], similar to other activity questionnaires [14].

#### CVD events

Any fatal or non-fatal CVD events, including coronary heart disease (myocardial infarction, non-myocardial infarction acute coronary syndrome), heart failure, and stroke (stroke, transient ischemic attack), were determined through participant contacts every 6 months, annual vital status searches, national death index searches every 5 years, telephone contact, and relevant medical records. Hospital records were sought for self-reported outcomes, and central adjudication was performed by trained physicians to ascertain CVD events. Two members of the CARDIA Endpoints Surveillance and Adjudication Subcommittee (ESAS) reviewed each record, applying standard outcome definitions to classify events; disagreements were resolved by the full CARDIA ESAS Committee.

#### Covariates

Age (years), race (Black or White), sex (male or female), smoking status (never, former, or current smoker), alcohol use (mL of alcohol consumed/day), educational attainment (highest grade of school completed), and family history of CVD (yes or no) were reported through questionnaire. Based on previous literature and causal directed acyclic graphs, these variables were considered potential confounders for the association between physical activity and CVD [11, 12]. Body mass index (BMI) was calculated based on measured height and weight at each examination. Blood pressure (hypertension) and fasting laboratory measures (glucose and cholesterol) were measured at each examination. BMI, hypertension, diabetes, and dyslipidemia were considered potential mediators for the association between physical activity and CVD based on previous literature and directed acyclic graphs [11, 12, 23].

### Statistical analysis

#### Summarizing physical activity

Physical activity trajectories were modeled among all CARDIA participants. A linear mixed model (LMM) for repeated measures of physical activity was used to generate concise summaries of exercise patterns over time [11, 12]. The physical activity slopes consisted of all observations of the physical activity scores before CVD event onset with the goal of using as much of the data per participant as possible and to stabilize the best linear unbiased predictions. The LMM had fixed effects for a four-level categorization of sex and race, continuous age, and their interactions, as well as random effects for participant and age, with unstructured covariance. We calculated expected physical activity level at age 18 and annual change for each participant from the fixed and random effects estimates the model provided. In order to capture the associations of lower level and faster decline in physical activity with increased CVD event risk and for easy interpretation, we changed the sign of both summaries.

#### Modeling the association of lower physical activity with incident CVD events

Unadjusted cumulative incidence of CVD events (coronary heart disease, congestive heart failure, and stroke) by sex and race/ethnicity was estimated using Kaplan-Meier methods [24]. We then expanded the data for each participant to include a record of each age between study entry and either metabolic disease onset, which was assumed to occur at the first visit at which it was detected, or at censoring by the end of the study of loss to follow-up. We used three sets of pooled logistic models to estimate the independent associations of the expected physical activity at age 18 and ensuing annual change with incidence of premature CVD events: adjusting for age (Model 1); adjusting for potential confounders including sex, race, family history of CVD, income, years of education, smoking status, and alcohol use (Model 2) [15]; and adjusting for all confounders in Model 2, as well as potential mediators, including BMI, hypertension, diabetes, and dyslipidemia (Model 3) [23]. Given the importance of age as a confounder for physical activity and CVD, we did not present unadjusted models. Smoking status, alcohol use, BMI, hypertension, diabetes, and dyslipidemia were time varying, with the last observation carried forward. We tested if sex and race modified the effect of physical activity (level and change) on incident premature CVD events. Pooled logistic models estimated the associations of various physical activity guideline thresholds (exceeding 2x minimum guidelines [> 600 EU], 1-2x minimum guidelines [300–600 EU], or below guidelines [< 300 EU]) at age 18 and through follow-up with onset of CVD events. We used Stata 16.0 (Statacorp, College Station, TX) for all analyses. Sensitivity analyses are listed in the Supplemental Appendix.

## Results

**Fig. 1:**
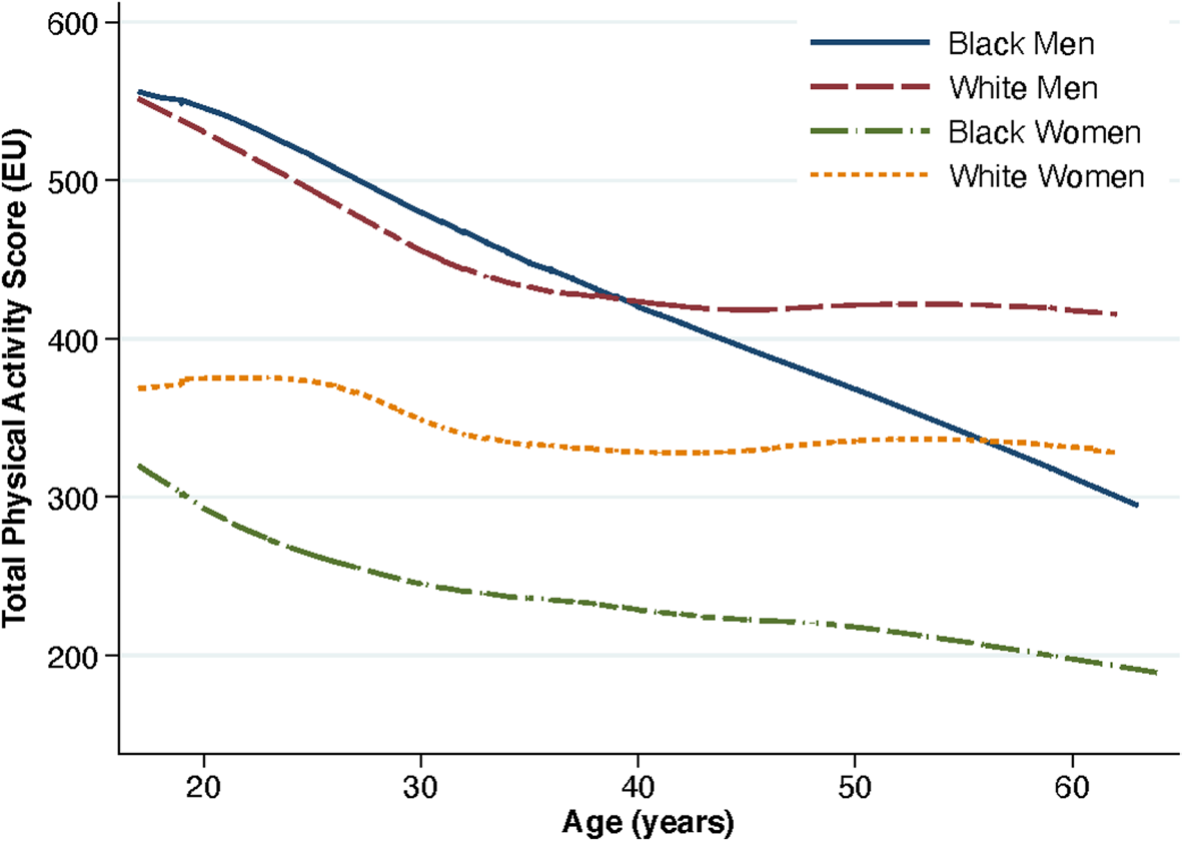
Average physical activity trajectories, by race and sex. Note: A total physical activity score of 300 exercise units (EU) approximates the Health and Human Services recommendations of approximately 150 minutes of moderate-intensity activity per week

Baseline demographic and health characteristic data of the 5114 participants (51.6% Black and 45.5% male) included in the sample are displayed in Table 1. Figure 1 portrays the decline in average physical activity from young adulthood in all race and sex groups. Supplemental Figs. 1-4 show the cumulative incidence of premature CVD events by race and sex.

**Table 1:** Baseline demographic and health characteristics of participants in the Coronary Artery Risk Development in Young Adults (CARDIA) study

	Total	White women	Black women	White men	Black men	
N	5114	1307	1480	1170	1157	
Baseline demographic characteristics	Median (IQR) / n (%)	Median (IQR) / n (%)	Median (IQR) / n (%)	Median (IQR) / n (%)	Median (IQR) / n (%)	*p*-value
Age (years)	25.0 (22.0–28.0)	26.0 (23.0–28.0)	24.0 (21.0–28.0)	26.0 (23.0–28.0)	24.0 (21.0–28.0)	< 0.001
Highest grade of school completed	13.0 (12.0–16.0)	15.0 (12.0–16.0)	13.0 (12.0–14.0)	15.0 (12.0–16.0)	12.0 (12.0–14.0)	< 0.001
Family history of cardiovascular disease	1022 (20.0%)	250 (19.1%)	310 (20.9%)	227 (19.4%)	235 (20.3%)	0.62
Body mass index (BMI)	23.4 (21.2–26.4)	22.0 (20.3–24.6)	24.2 (21.2–28.9)	23.7 (21.9–26.0)	23.7 (21.7–26.4)	< 0.001
< 25 kg/m^2^	3328 (65.3%)	1012 (77.7%)	823 (55.8%)	761 (65.2%)	732 (63.5%)	
25–30 kg/m^2^	1170 (23.0%)	195 (15.0%)	337 (22.8%)	334 (28.6%)	304 (26.4%)	
> 30 kg/m^2^	599 (11.8%)	95 (7.3%)	315 (21.4%)	72 (6.2%)	117 (10.1%)	
Smoking status						< 0.001
Never	2856 (56.2%)	685 (52.7%)	885 (60.1%)	670 (57.8%)	616 (53.8%)	
Former	676 (13.3%)	261 (20.1%)	127 (8.6%)	182 (15.7%)	106 (9.3%)	
Current	1546 (30.4%)	355 (27.3%)	461 (31.3%)	307 (26.5%)	423 (36.9%)	
Alcohol (mL of alcohol consumed per day)	5.4 (0.9–15.5)	4.8 (0.9–12.1)	1.8 (0.0–6.9)	11.1 (3.7–23.2)	10.2 (2.0–25.2)	< 0.001
Total physical activity score at enrollment (EU)	360.0 (197.0–578.0)	351.0 (207.0–543.0)	228.0 (103.0–396.0)	462.0 (288.0–672.0)	472.0 (271.0–723.0)	< 0.001
Total physical activity score at age 18 (EU)	363.1 (241.1–534.1)	333.3 (235.0–473.4)	238.1 (160.5–333.6)	466.1 (327.8–612.0)	515.7 (366.5–681.9)	< 0.001
Annual reduction in total physical activity score (EU)	2.4 (0.4–5.1)	1.5 (−0.4–3.5)	1.6 (0.1–3.5)	2.5 (0.3–4.9)	5.7 (3.3–8.2)	< 0.001
Expected total physical activity at age 18						
< 300 EU	1915 (37.5%)	545 (41.7%)	989 (67.0%)	231 (19.8%)	150 (13.0%)	< 0.001
300–600 EU	2274 (44.6%)	633 (48.5%)	432 (29.2%)	635 (54.4%)	574 (49.8%)	
> 600 EU	913 (17.9%)	128 (9.8%)	56 (3.8%)	301 (25.8%)	428 (37.2%)	
Always meeting total physical activity level from young adulthood to middle age						
< 300 EU	2128 (41.7%)	589 (45.1%)	1067 (72.2%)	263 (22.5%)	209 (18.1%)	< 0.001
300–600 EU	2202 (43.2%)	612 (46.9%)	362 (24.5%)	646 (55.4%)	582 (50.5%)	
> 600 EU	772 (15.1%)	105 (8.0%)	48 (3.2%)	258 (22.1%)	361 (31.3%)	

*IQR* Interquartile range; *EU* Exercise units

A total physical activity score of 300 exercise units (EU) approximates the Health and Human Services recommendations of approximately 150 minutes of moderate-intensity activity per week

Pooled logistic regression model estimates for the associations between premature CVD event incidence and the two physical activity summaries (physical activity level at age 18 and subsequent declines in physical activity) are shown in Table 2. Model 1 adjusted for age only whereas Model 2 adjusted for race, sex, education, income, family history of CVD, smoking status, and alcohol in addition to age. Model 2, the fully adjusted model, indicates the association between lower physical activity score (per 100 units) at age 18 and the higher odds of incident CHD (14%), heart failure (21%), stroke (20%), and any CVD (15%). Each additional annual 1 EU reduction in the physical activity score was associated with a higher annual odds of incident heart failure (7%), stroke (6%), and any CVD (4%). Sex and race categories did not modify the effect of physical activity (level and change) on incident CVD events (all *p* > 0.05). Sensitivity analyses are shown in the Supplemental Appendix.

**Table 2:** Associations between physical activity trajectories and incidence of premature cardiovascular disease (CVD) events in the CARDIA study

	Model 1 (adjusted for age)^a^			Model 2 (fully adjusted)^b^			Model 3 (fully adjusted with mediators)^c^					
	OR	95% CI	p	OR	95% CI	p	OR	95% CI	p	% Mediation	95% CI	p
**Any coronary heart disease (CHD) - fatal or nonfatal** (myocardial infarction, non-myocardial infarction acute coronary syndrome)												
Lower physical activity score (per 100 Exercise Units) at age 18	1.04	0.95, 1.14	0.400	**1.14**	**1.02, 1.28**	**0.025**	1.08	0.96, 1.21	0.200	**43%**	**4, 82%**	**0.029**
Annual reduction in total physical activity score (per 1 Exercise Unit)	**1.05**	**1.02, 1.09**	**0.004**	1.03	0.99, 1.07	0.160	1.01	0.97, 1.05	0.760	78%	−35, 191%	0.17
**Any heart failure - fatal or nonfatal** (congestive heart failure)												
Lower physical activity score (per 100 Exercise Units) at age 18	**1.26**	**1.10, 1.45**	**< 0.001**	**1.21**	**1.05, 1.38**	**0.008**	1.13	1.00, 1.28	0.058	**35%**	**11, 58%**	**0.004**
Annual reduction in total physical activity score (per 1 Exercise Unit)	**1.17**	**1.10, 1.24**	**< 0.001**	**1.07**	**1.02, 1.13**	**0.011**	1.04	0.99, 1.10	0.120	**38%**	**3, 72%**	**0.032**
**Any stroke - fatal or nonfatal** (stroke, transient ischemic attack)												
Lower physical activity score (per 100 Exercise Units) at age 18	**1.32**	**1.16, 1.50**	**< 0.001**	**1.20**	**1.04, 1.39**	**0.013**	1.14	0.99, 1.31	0.07	**30%**	**6, 53%**	**0.013**
Annual reduction in total physical activity score (per 1 Exercise Unit)	**1.14**	**1.07, 1.21**	**< 0.001**	**1.06**	**1.00, 1.13**	**0.040**	1.04	0.98, 1.10	0.180	35%	−2, 73%	0.06
**Any CVD - fatal or nonfatal** (myocardial infarction, coronary revascularization, non-NI acute coronary syndrome, congestive heart failure, stroke, transient ischemic attack, carotid artery disease, peripheral artery disease, abdominal aortic aneurysm)												
Lower physical activity score (per 100 Exercise Units) at age 18	**1.12**	**1.05, 1.20**	**< 0.001**	**1.15**	**1.06, 1.24**	**< 0.001**	**1.08**	**1.00, 1.17**	**0.037**	**41%**	**17, 66%**	**< 0.001**
Annual reduction in total physical activity score (per 1 Exercise Unit)	**1.08**	**1.05, 1.11**	**< 0.001**	**1.04**	**1.01, 1.07**	**0.011**	1.02	0.99, 1.04	0.300	**59%**	**10, 108%**	**0.019**

Note: Boldface indicates statistical significance (*p* < 0.05)

^a^Model 1 includes: physical activity level at age 18, annual reduction in physical activity, age. Separate models are presented for each outcome (CHD, heart failure, stroke, CVD)

^b^Model 2 includes: physical activity level at age 18, annual reduction in physical activity, age, race, sex, education, income, family history of CVD, smoking status, and alcohol. Separate models are presented for each outcome (CHD, heart failure, stroke, CVD)

^c^Model 3 includes: physical activity level at age 18, annual reduction in physical activity, age, race, sex, education, income, family history of CVD, smoking status, alcohol, body mass index, hypertension, diabetes, and dyslipidemia. Separate models are presented for each outcome (CHD, heart failure, stroke, CVD)

In models including potential mediators such as BMI, hypertension, diabetes, and dyslipidemia (Table 2, Model 3), the association between physical activity and incident CVD events was attenuated. In particular, these factors mediated 41% of the total effect of physical activity at age 18 and 59% of the total effect of the annual reduction in physical activity on incident CVD events. When adding each potential mediator (BMI, hypertension, diabetes, and dyslipidemia) to the model separately (Appendix D), diabetes accounted for the highest percent of the total effect mediated (24% for physical activity at age 18 and 38% for annual reduction in physical activity).

Table 3 shows associations with various physical activity guideline thresholds (> 600 EU or 300–600 EU vs < 300 EU) at age 18 and through follow-up with onset of CVD events. Meeting the minimum HHS physical activity guidelines (300–600 EU, OR 0.69) and twice the minimum HHS guidelines (> 600 EU, OR 0.60) at age 18 was associated with lower odds of any premature CVD events compared to not meeting the minimum HHS guidelines. Meeting the minimum HHS physical activity guidelines (OR 0.74) and twice the minimum HHS guidelines (OR 0.55) from young adulthood to middle age (estimated physical activity level from linear mixed model was always above the threshold over the 30-year follow-up period) was associated with lower odds of any premature CVD events compared to not meeting the minimum HHS guidelines.

**Table 3:** Associations between meeting the Department of Health and Human Services physical activity guidelines at age 18 and through the follow-up period and onset of cardiovascular disease events in the CARDIA study

	Any CHD			Any CHF			Any Stroke			Any CVD		
	OR	95% CI	p	OR	95% CI	P	OR	95% CI	p	OR	95% CI	p
Expected total physical activity at age 18^a^												
< 300 EU	reference			reference			reference			reference		
300–600 EU	**0.50**	**0.34, 0.72**	**< 0.001**	1.08	0.64, 1.82	0.770	0.69	0.42, 1.15	0.150	**0.69**	**0.52, 0.91**	**0.010**
> 600 EU	**0.59**	**0.38, 0.93**	**0.024**	0.60	0.27, 1.32	0.200	0.51	0.23,1.14	0.100	**0.60**	**0.41, 0.88**	**0.008**
Always meeting total physical activity level from young adulthood to middle age^a^												
< 300 EU	reference			reference			reference			reference		
> 300 EU	**0.60**	**0.42, 0.85**	**0.004**	0.82	0.51, 1.32	0.420	0.74	0.46, 1.18	0.210	**0.74**	**0.57, 0.96**	**0.021**
> 600 EU	0.68	0.39, 1.19	0.180	**0.23**	**0.05, 0.99**	**0.048**	0.45	0.15, 1.36	0.160	**0.55**	**0.34, 0.91**	**0.019**

A total physical activity score of 300 exercise units (EU) approximates the Health and Human Services recommendations of approximately 150 minutes of moderate-intensity activity per week. Always meeting total physical activity level from young adulthood to middle age indicates that the estimated physical activity level from the linear mixed model was always above the threshold over the 30-year follow-up period

^a^Covariates: age, race, sex, education, income, family history of cardiovascular disease, smoking status, and alcohol

## Discussion

In this prospective cohort study with 30-years of follow-up, we found that a high level of physical activity during young adulthood is a crucial starting point for preventing premature CVD events before age 60. Physical activity in young adults is associated with lower incidence of premature CVD events, specifically heart failure and stroke events, independent of physical activity later in adulthood. In addition, for any given young adult physical activity set-point, decline in physical activity through the adult life course is also associated with incident premature heart failure and stroke events. Thus, it is important to maintain high levels of physical activity throughout the adult life course. While meeting the minimum HHS physical activity guidelines of 150 minutes of moderate-intensity physical activity per week at age 18 and through the adult life course was protective of premature CVD events, meeting twice the minimum HHS guidelines was even more protective of premature CVD events.

Our study builds upon prior research linking physical activity to subsequent CVD events [2–4], but expands on this literature by examining trajectories with 30-years of follow-up from young to middle adulthood and also examining premature CVD events prior to age 60. The 2018 HHS Physical Activity Guidelines Scientific Advisory Committee concluded there was insufficient evidence that the association between physical activity and CVD events varied by age group, with a particular evidence gap for young adults [5]. We found physical activity levels in young adulthood are protective of premature heart failure and stroke events. Examining the various guideline thresholds, we found meeting both the current recommended minimum levels and twice the recommended minimum levels of moderate physical activity at age 18 and through midlife were protective of premature CVD events before age 60.

Several potential mechanisms may explain the link between physical activity and fewer premature CVD events. We found, collectively, that diabetes, BMI, hypertension, and dyslipidemia mediate 41–59% of the association between physical activity and CVD events, with diabetes as the largest contributor. Physical activity may improve inflammatory and metabolic factors which could protect from premature CVD [23]. High levels of physical activity in young and middle adulthood are protective of incident hypertension [11], which could be particularly implicated in the prevention of stroke events. It is notable that both physical activity level and slope were independently associated with premature heart failure and stroke event onset, even after adjusting for several potential sociodemographic and behavioral confounders.

Our findings indicate that young adult physical activity levels provide protection from subsequent premature CVD events, independent of physical activity levels up through midlife. Therefore, young adults may be an important target for interventions to ensure adequate physical activity levels. We find that physical activity declines, on average, during young adulthood in all race and sex groups, consistent with previous literature [6–8]. Educational, economic, and social transitions such as starting college, entering the workforce, or becoming parents during young adulthood may lead to fewer opportunities for organized or leisure time physical activity [25, 26].

Disparities in physical activity and premature CVD events across race and sex are noteworthy over the 30-year follow-up period. Black women reported the lowest physical activity levels from young adulthood through midlife. While young men start with high average physical activity levels, these levels persistently decline through young adulthood, with further declines among Black men through midlife, similar to previously reported patterns [21, 27]. In terms of disparities in premature CVD events, we found that Black populations had the highest incidence of premature heart failure and stroke events while men had the highest incidence of premature CHD events, similar to known race disparities in CVD including CHD and stroke [1, 28, 29]. Despite these disparities, we did not find evidence of effect modification by race or sex in the association between physical activity and premature CVD events. This finding of no effect modification by race or sex also contributes to an HHS-articulated gap for the Physical Activity Guidelines for Americans Scientific Report [5]. The benefits of physical activity to prevent premature CVD do not differ by race or sex which indicates that disparities in premature CVD events are likely due to other factors such as social determinants of health.

### Clinical perspectives

Clinicians should assess for and promote physical activity at health maintenance visits. Despite evidence of the feasibility, validity, and effectiveness of assessing and promoting physical activity in clinical settings, physical activity is less routinely assessed in clinical practice compared to smoking, obesity, blood pressure, glucose, and lipid profiles [30], though CVD risk associated with physical inactivity is comparable to smoking [31]. Messaging promoting optimizing health, rather than the need to avoid risk, may better evoke responses from young adults [32]. Young adults may be more prone to taking on immediate risks (e.g. not being physically active) if they believe the outcome related to the counseling is far in the future; this is an instance of the concept referred to as temporal discounting [33]. The urgency of high levels of physical activity in young adulthood is emphasized by our finding that physical activity may prevent premature CVD events, including death before age 60.

Physical activity interventions at universities [34], workplaces [35], community organizations [36, 37], and faith-based organizations [38] may promote physical activity among young and middle-aged adults. Digital interventions including social media, text messages, e-mails, games, and multicomponent interventions, may hold future promise for promoting physical activity especially among young adults [39]; however, further research on and development of digital interventions for promoting physical activity is required.

### Limitations and strengths

The CARDIA Physical Activity History does not directly quantify duration and frequency within activity categories and was based on self-reported data, which may be subject to information bias, including recall (12-month recall) and prevarication bias. Social desirability bias may lead to inflation of the self-reported physical activity and limit generalizability. Nonetheless, repeated measures of the same physical activity measure were collected in 2–5-year intervals across the 30-year follow-up period, which is a unique strength. The sampling design of CARDIA was not representative of all races or ethnicities in the US, which may limit generalizability; however the study specifically focused on participants identifying as Black or White race [13]. While we adjusted for several potential confounders including age, sex, race, education, family history, smoking status, and alcohol, there is the possibility of unmeasured confounders. Given the small proportion of the sample with premature CVD events by age 60, we may have been underpowered to detect associations with some of the individual premature CVD event outcomes, particularly with the guideline threshold analyses. Future research examining these outcomes later in adulthood, when CVD event outcomes become more common, may have greater power to inform various guideline level thresholds.

## Conclusion

In conclusion, low physical activity levels in young adulthood and declining physical activity in later adulthood are each significantly and independently associated with premature CVD events before age 60. Clinicians and public health programs should emphasize, prioritize, and develop interventions to promote physical activity across the lifespan, including young adulthood, which is an important time when individuals establish physical activity lifestyle behaviors for the rest of their life course. Regardless of young adult physical activity level, sustained or increased physical activity later in midlife can also prevent premature CVD events and death.

## Supplementary Information

**Additional file 1.**

## Data Availability

Data from the Coronary Artery Risk Development in Young Adults Study may be accessed through a manuscript proposal or ancillary study proposal (details at https://www.cardia.dopm.uab.edu/invitation-to-new-investigators).

## Abbreviations

BMI: Body mass index
CHD: Coronary heart disease
CVD: Cardiovascular disease
CARDIA: Coronary Artery Risk Development in Young Adults Study
EU: Exercise units
HHS: Department of Health and Human Services
LMM: Linear mixed model
MET: Metabolic equivalent
US: United States
