# Supplementary material for: Physical activity from young adulthood to middle age and premature cardiovascular disease events: a 30-year population-based cohort study
Source: Int J Behav Nutr Phys Act. 2022 Sep 20;19:123. doi: 10.1186/s12966-022-01357-2 (PMC9487136; doi:10.1186/s12966-022-01357-2)

**Supplemental Appendix**

| Appendix A. Associations between physical activity trajectories, with annual reduction in physical activity before age 35, and incidence of premature cardiovascular disease (CVD) events in the CARDIA study | | | |
| --- | --- | --- | --- |
|  | OR | 95% CI | p |
| **Any coronary heart disease (CHD) - fatal or nonfatal** (myocardial infarction, non-myocardial infarction acute coronary syndrome) | | | |
| Lower physical activity score (per 100 Exercise Units) at age 18 | **1.21** | **1.08, 1.37** | **0.002** |
| Annual reduction in total physical activity score, age <35 (per 1 Exercise Unit) | **1.07** | **1.03, 1.11** | **<0.001** |
| **Any heart failure - fatal or nonfatal** (congestive heart failure) | | | |
| Lower physical activity score (per 100 Exercise Units) at age 18 | 1.14 | 1.00, 1.29 | 0.056 |
| Annual reduction in total physical activity score, age <35 (per 1 Exercise Unit) | 1.01 | 0.96, 1.05 | 0.820 |
| **Any Stroke - fatal or nonfatal** (stroke, transient ischemic attack) | | | |
| Lower physical activity score (per 100 Exercise Units) at age 18 | 1.11 | 0.98, 1.25 | 0.090 |
| Annual reduction in total physical activity score, age <35 (per 1 Exercise Unit) | 0.98 | 0.94, 1.02 | 0.280 |
| **Any CVD - fatal or nonfatal** (myocardial infarction, coronary revascularization, non-NI acute coronary syndrome, congestive heart failure, stroke, transient ischemic attack, carotid artery disease, peripheral artery disease, abdominal aortic aneurysm) | | | |
| Lower physical activity score (per 100 Exercise Units) at age 18 | **1.14** | **1.06, 1.23** | **<0.001** |
| Annual reduction in total physical activity score, age <35 (per 1 Exercise Unit) | 1.02 | 1.00, 1.04 | 0.120 |
| Note: Boldface indicates statistical significance (p<0.05). | | | |
| Model includes: physical activity level at age 18, annual reduction in physical activity age <35, age, race, sex, education, income, family history of CVD, smoking status, and alcohol. Separate models are presented for each outcome (CHD, heart failure, stroke, CVD). | | | |

| Appendix B. Associations between physical activity trajectories, with annual reduction in physical activity after age 35, and incidence of premature cardiovascular disease (CVD) events in the CARDIA study | | | |  |
| --- | --- | --- | --- | --- |
|  | Fully adjusted^a^ | | | |
|  | OR | 95% CI | p | |
| **Any coronary heart disease (CHD) - fatal or nonfatal** (myocardial infarction, non-myocardial infarction acute coronary syndrome) | | | |  |
| Lower physical activity score (per 100 Exercise Units) at age 18 | **1.18** | **1.06, 1.33** | **0.004** | |
| Annual reduction in total physical activity score, age ≥35 (per 1 Exercise Unit) | **1.08** | **1.03, 1.14** | **0.003** | |
| **Any heart failure - fatal or nonfatal** (congestive heart failure) | | | |  |
| Lower physical activity score (per 100 Exercise Units) at age 18 | **1.33** | **1.15, 1.54** | **<0.001** | |
| Annual reduction in total physical activity score, age ≥35 (per 1 Exercise Unit) | **1.22** | **1.16, 1.29** | **<0.001** | |
| **Any Stroke - fatal or nonfatal** (stroke, transient ischemic attack) | | | |  |
| Lower physical activity score (per 100 Exercise Units) at age 18 | **1.26** | **1.11, 1.43** | **<0.001** | |
| Annual reduction in total physical activity score, age ≥35 (per 1 Exercise Unit) | **1.17** | **1.10, 1.24** | **<0.001** | |
| **Any CVD - fatal or nonfatal** (myocardial infarction, coronary revascularization, non-NI acute coronary syndrome, congestive heart failure, stroke, transient ischemic attack, carotid artery disease, peripheral artery disease, abdominal aortic aneurysm) | | | |  |
| Lower physical activity score (per 100 Exercise Units) at age 18 | **1.20** | **1.12, 1.29** | **<0.001** | |
| Annual reduction in total physical activity score, age ≥35 (per 1 Exercise Unit) | **1.13** | **1.09, 1.17** | **<0.001** | |
| Note: Boldface indicates statistical significance (p<0.05). | | | |  |
| ^a^Model includes: physical activity level at age 18, annual reduction in physical activity age ≥35, age, race, sex, education, income, family history of CVD, smoking status, and alcohol. Separate models are presented for each outcome (CHD, heart failure, stroke, CVD). | | | |  |

| Appendix C. Associations between physical activity trajectories and incidence of premature cardiovascular disease (CVD) events in the CARDIA study, adjusting for last recorded physical activity | | | | |
| --- | --- | --- | --- | --- |
|  | OR | 95% CI | p |  |
| **Any coronary heart disease (CHD) - fatal or nonfatal** (myocardial infarction, non-myocardial infarction acute coronary syndrome) | | | | |
| Lower physical activity score (per 100 Exercise Units) at age 18 | **1.45** | **1.21, 1.73** | **<0.001** |  |
| Lower physical activity score (per 100 Exercise Units), last recorded | **0.81** | **0.71, 0.93** | **0.002** |  |
| Annual reduction in total physical activity score (per 1 Exercise Unit) | **1.15** | **1.06, 1.25** | **<0.001** |  |
| **Any heart failure - fatal or nonfatal** (congestive heart failure) | | | | |
| Lower physical activity score (per 100 Exercise Units) at age 18 | **1.33** | **1.04, 1.71** | **0.025** |  |
| Lower physical activity score (per 100 Exercise Units), last recorded | 0.92 | 0.76, 1.11 | 0.37 |  |
| Annual reduction in total physical activity score (per 1 Exercise Unit) | **1.12** | **1.01, 1.26** | **0.041** |  |
| **Any Stroke - fatal or nonfatal** (stroke, transient ischemic attack) | | | | |
| Lower physical activity score (per 100 Exercise Units) at age 18 | **1.76** | **1.38, 2.25** | **<0.001** |  |
| Lower physical activity score (per 100 Exercise Units), last recorded | **0.73** | **0.62, 0.86** | **<0.001** |  |
| Annual reduction in total physical activity score (per 1 Exercise Unit) | **1.27** | **1.14, 1.43** | **<0.001** |  |
| **Any CVD - fatal or nonfatal** (myocardial infarction, coronary revascularization, non-NI acute coronary syndrome, congestive heart failure, stroke, transient ischemic attack, carotid artery disease, peripheral artery disease, abdominal aortic aneurysm) | | | | |
| Lower physical activity score (per 100 Exercise Units) at age 18 | **1.47** | **1.29, 1.67** | **<0.001** |  |
| Lower physical activity score (per 100 Exercise Units), last recorded | **0.81** | **0.74, 0.89** | **<0.001** |  |
| Annual reduction in total physical activity score (per 1 Exercise Unit) | **1.17** | **1.10, 1.24** | **<0.001** |  |
| Note: Boldface indicates statistical significance (p<0.05). | | | | |
| Model includes: physical activity level at age 18, last recorded physical activity level (30-year follow-up or last physical activity level prior to a CVD event), annual reduction in physical activity, age, race, sex, education, income, family history of CVD, smoking status, and alcohol. Separate models are presented for each outcome (CHD, heart failure, stroke, CVD). | | | | |

| Appendix D. Potential mediators of the association between physical activity trajectories and premature cardiovascular disease (CVD) events in the CARDIA study | | | | | | | | | | | | | | | |
| --- | --- | --- | --- | --- | --- | --- | --- | --- | --- | --- | --- | --- | --- | --- | --- |
|  | Any coronary heart disease (CHD) | | |  | Any heart failure | | |  | Any Stroke | | |  | Any CVD | | |
|  | % mediation | 95% CI | p |  | % mediation | 95% CI | p |  | % mediation | 95% CI | p |  | % mediation | 95% CI | p |
| **Model with BMI** |  |  |  |  |  |  |  |  |  |  |  |  |  |  |  |
| Lower physical activity score (per 100 Exercise Units) at age 18 | 18% | -1%, 38% | 0.07 |  | 11% | 1%, 23% | 0.070 |  | 5% | -4%, 13% | 0.290 |  | **14%** | **3%, 24%** | **0.01** |
| Annual reduction in total physical activity score (per 1 Exercise Unit) | 40% | -20%, 100% | 0.19 |  | 14% | -2%, 30% | 0.090 |  | 8% | -7%, 23% | 0.280 |  | **25%** | **3%, 47%** | **0.028** |
| **Model with hypertension** |  |  |  |  |  |  |  |  |  |  |  |  |  |  |  |
| Lower physical activity score (per 100 Exercise Units) at age 18 | 15% | -1%, 31% | 0.060 |  | 10% | 0%, 20% | 0.054 |  | **18%** | **2%, 34%** | **0.031** |  | **17%** | **4%, 29%** | **0.008** |
| Annual reduction in total physical activity score (per 1 Exercise Unit) | 28% | 15%, 71% | 0.2 |  | 8% | -3%, 20% | 0.170 |  | 19% | -3%, 40% | 0.080 |  | **21%** | **3%, 47%** | **0.028** |
| **Model with diabetes** |  |  |  |  |  |  |  |  |  |  |  |  |  |  |  |
| Lower physical activity score (per 100 Exercise Units) at age 18 | **22%** | **2%, 43%** | **0.035** |  | **22%** | **7%, 38%** | **0.004** |  | **20%** | **5%, 36%** | **0.011** |  | **24%** | **9%, 38%** | **0.002** |
| Annual reduction in total physical activity score (per 1 Exercise Unit) | 43% | -21%, 107% | 0.19 |  | **26%** | **3%, 50%** | **0.028** |  | 28% | -1%, 56% | 0.057 |  | **38%** | **5%, 71%** | **0.024** |
| **Model with dyslipidemia (high triglycerides or low HDL)** |  |  |  |  |  |  |  |  |  |  |  |  |  |  |  |
| Lower physical activity score (per 100 Exercise Units) at age 18 | 15% | 0%, 31% | 0.053 |  | 4% | -2%, 10% | 0.17 |  | 3% | -2%, 9% | 0.28 |  | **11%** | **3%, 19%** | **0.009** |
| Annual reduction in total physical activity score (per 1 Exercise Unit) | 27% | 11%, 66% | 0.17 |  | 5% | -2%, 11% | 0.170 |  | 4% | -3%, 12% | 0.270 |  | **15%** | **2%, 29%** | **0.027** |
| Note: Boldface indicates statistical significance (p<0.05). | | | | | | | | | | | |  |  |  |  |
| Model 1 includes: physical activity level at age 18, annual reduction in physical activity, age. Separate models are presented for each outcome (CHD, heart failure, stroke, CVD). | | | | | | | | | | | |  |  |  |  |
| Model 2 includes: physical activity level at age 18, annual reduction in physical activity, age, race, sex, education, family history of CVD, smoking status, alcohol, and body mass index. Separate models are presented for each outcome (CHD, heart failure, stroke, CVD). | | | | | | | | | | | |  |  |  |  |

Supplemental Figure 1. Any CVD incidence, by race and sex


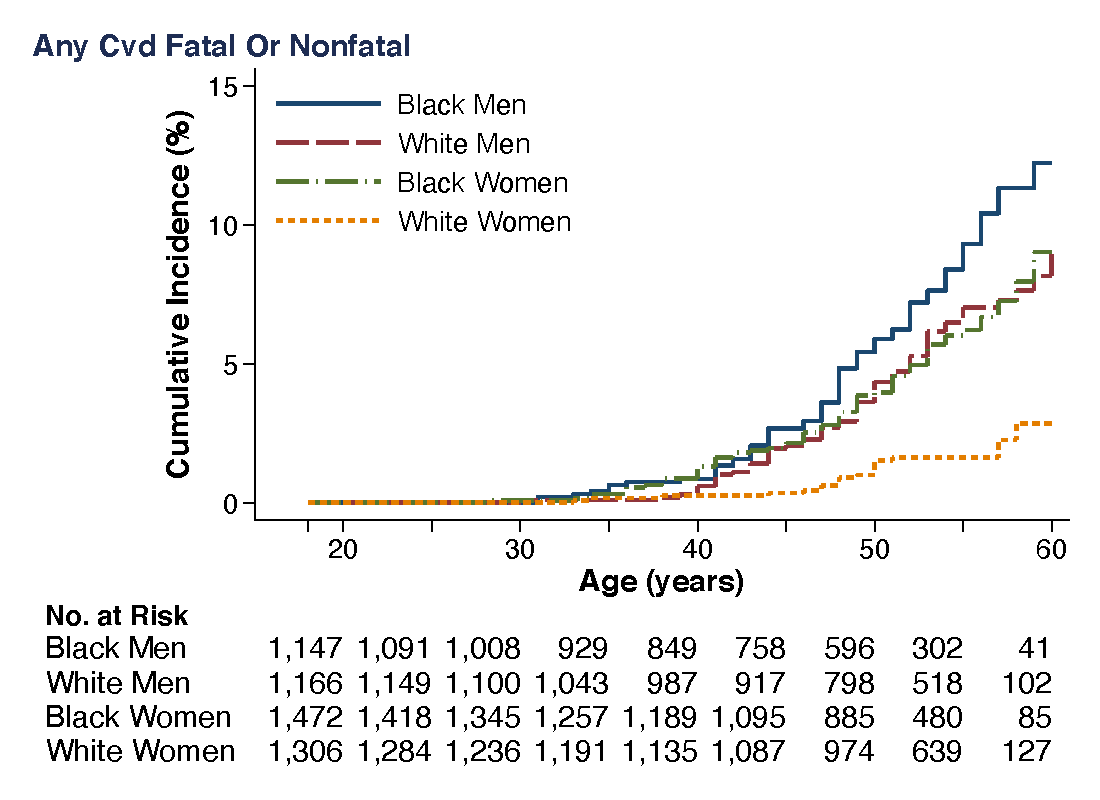


Supplemental Figure 2. Any CHD incidence, by race and sex


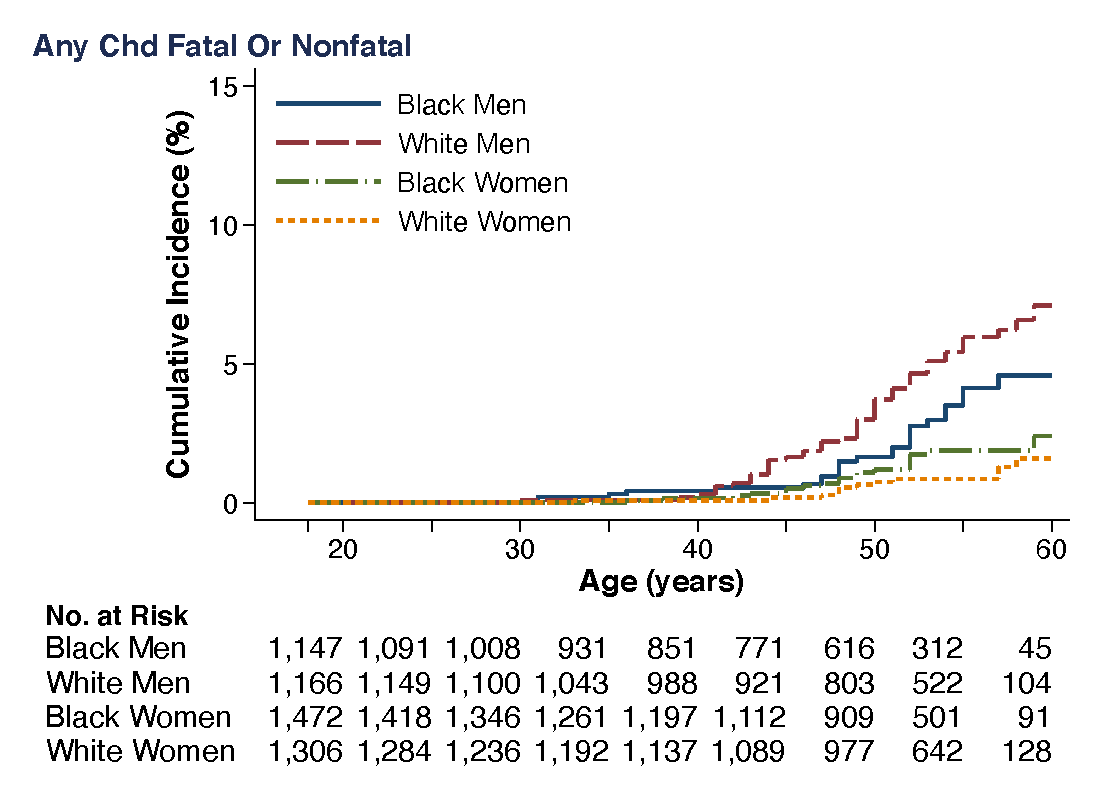


Supplemental Figure 3. Any heart failure incidence, by race and sex


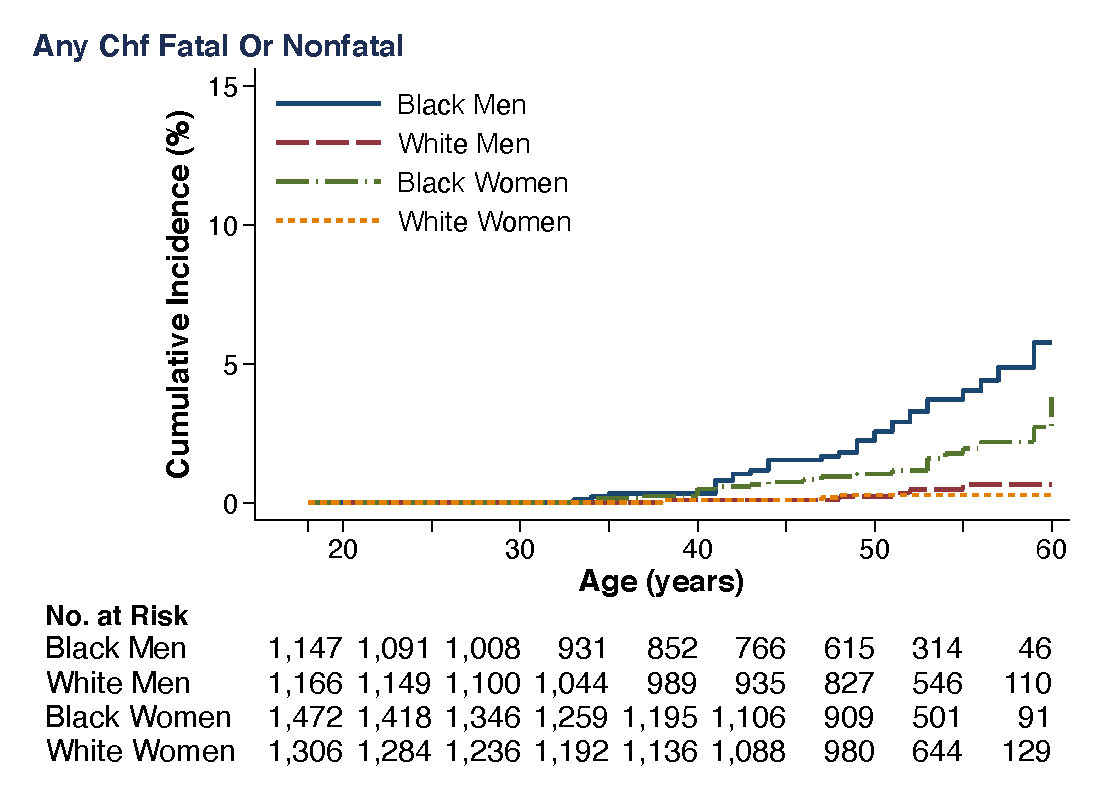


Supplemental Figure 4. Any stroke incidence, by race and sex


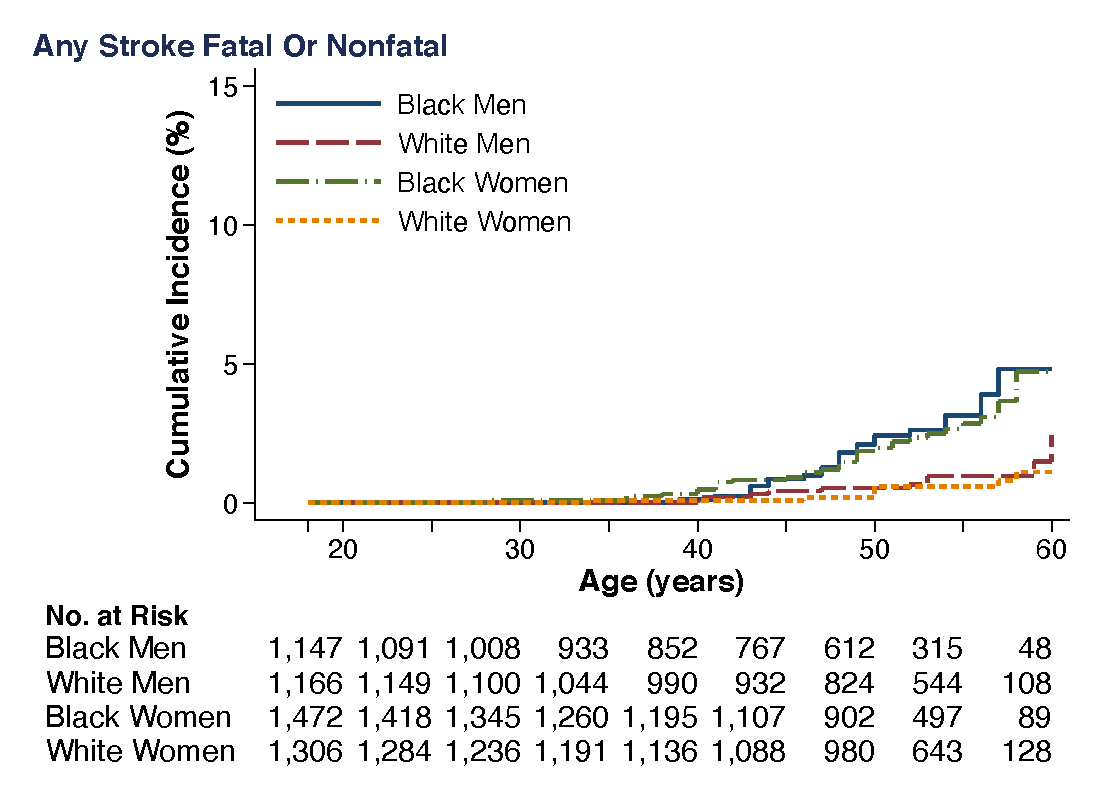

Supplement: Supplementary file 1 — Additional file 1. [file 12966_2022_1357_MOESM1_ESM.docx]
